# THE ASSOCIATION OF COVID-19 OUTBREAKS AND OTHER FACTORS WITH NURSING HOME RESIDENTS’ QUALITY OF LIFE

**DOI:** 10.1093/geroni/igad104.1114

**Published:** 2023-12-21

**Authors:** Matthias Hoben, Emily Dymchuk, Sube Banerjee, Carole Estabrooks

**Affiliations:** York University, Toronto, Ontario, Canada; University of Alberta, Edmonton, Alberta, Canada; University of Plymouth, Plymouth, England, United Kingdom; University of Alberta, Edmonton, Alberta, Canada

## Abstract

Nursing home (NH) residents’ Quality of life (QoL) is an important goal of care. However, it is understudied, particularly during the COVID-19 pandemic. Our objective was to examine whether COVID-19 outbreaks, care aide emotional exhaustion, and lack of resident access to geriatric professionals were associated with NH residents’ QoL. In this cross-sectional study (Jul-Dec 2021), we purposefully selected 9 NHs in Alberta, Canada based on their COVID-19 exposure (no or minor/short outbreaks vs repeated or extensive outbreaks). Using a validated questionnaire (DEMQOL-CH), we assessed dementia-specific QoL of 689 residents from 18 care units through video-based interviews with care aides. Independent variables included COVID-19 outbreak in the NH in the last 2 weeks (health authority records), proportion of care aides on a care unit with high emotional exhaustion scores (9-item short form Maslach Burnout Inventory), and resident access to geriatric professionals (validated facility survey). We ran mixed-effects regression models, adjusted for facility and care unit characteristics (validated facility and care unit surveys), and resident covariates (Resident Assessment Instrument – Minimum Data Set 2.0). COVID-19 outbreaks within two weeks of the data collection (β=0.189, 95% confidence interval [CI]: 0.058;0.320), higher proportions of emotionally exhausted care aides on a care unit (β=0.681, 95%CI: 0.246;1.115) and lack of access to geriatric professionals (β=0.216, 95%CI: 0.003;0.428) were significantly associated with poorer resident QoL. Policies aimed at reducing infection outbreaks, better supporting care staff, and increasing access to geriatric specialists, may help to mitigate negative effects of COVID-19 NH residents’ QoL.

